# CircRNAs: a new target for the diagnosis and treatment of digestive system neoplasms

**DOI:** 10.1038/s41419-021-03495-0

**Published:** 2021-02-24

**Authors:** Jie Li, Qiang Xu, Zi-jian Huang, Ning Mao, Zhi-tao Lin, Long Cheng, Bei Sun, Gang Wang

**Affiliations:** 1 Department of Pancreatic and Biliary Surgery, The First Affiliated Hospital of Harbin Medical University, Harbin, Heilongjiang Province China; 2 Key Laboratory of Hepatosplenic Surgery, Ministry of Education, The First Affiliated Hospital of Harbin Medical University, Harbin, China; 3 Department of General Surgery, Peking Union Medical College Hospital, Chinese Academy of Medial Sciences, Beijing, 100730 China

**Keywords:** Tumour biomarkers, Non-coding RNAs

## Abstract

A circRNA is a type of endogenous noncoding RNA that consists of a closed circular RNA molecule formed by reverse splicing; these RNAs are widely distributed in a variety of biological cells. In contrast to linear RNAs, circRNAs have no 5′ cap or 3′ poly(A) tail. They have a stable structure, a high degree of conservation, and high stability, and they are richly and specifically expressed in certain tissues and developmental stages. CircRNAs play a very important role in the occurrence and progression of malignant tumors. According to their origins, circRNAs can be divided into four types: exon-derived circRNAs (ecRNAs), intron-derived circRNAs (ciRNAs), circRNAs containing both exons and introns (EIciRNAs) and intergenic circRNAs. A large number of studies have shown that circRNAs have a variety of biological functions, participate in the regulation of gene expression and play an important role in the occurrence and progression of tumors. In this paper, the structure and function of circRNAs are reviewed, along with their biological role in malignant tumors of the digestive tract, in order to provide a reference for the diagnosis and treatment of digestive system neoplasms.

## Facts

- circRNA is an endogenous non-coding RNA with a stable structure, high degree of conservation and stability.
- circRNA can be used as an important molecular marker and potential therapeutic target for the early diagnosis of digestive tract tumors.
- Activating and blocking the expression of circRNA will effectively regulate the disease process of tumors and will provide a new therapeutic strategy.

## Open questions

- How is circRNA normally degraded in cells?
- Is there a synergistic or antagonistic relationship between the different regulatory pathways of circRNA?
- How can the binding process of circRNA and miRNA be accurately blocked?
- How to detect circRNA more accurately and economically?

## Introduction

Sanger’s team discovered circRNAs in 1976, but they were considered at the time to be RNA molecules formed by miscutting; as a result, they did not attract much attention at first^1^. However, with the continuous development of RNA sequencing technology and bioinformatics, an increasing number of circRNAs have been found, and circRNA has gradually been recognized to play an important role in regulating the biological processes of eukaryotes. CircRNAs, a type of closed circular noncoding RNAs that are ubiquitous in organisms, can resist hydrolysis by exonucleases and participate in the regulation of gene expression, which is closely related to the occurrence and progression of many diseases^2^. In contrast to the way linear RNA is generated, circRNA is formed by “reverse splicing” in a nonclassical way. The vast majority of circRNAs consist of 1–5 exons of a linear messenger RNA (mRNA) coding region with the 3′ and 5′ ends connected by “reverse splicing” to form a continuous covalent ring structure, sometimes including intron regions as well^3^. In addition, circRNAs can come from introns, long noncoding RNAs (lncRNAs), reverse transcripts, or intergenic regions^4^. Based on differences in origin and sequence composition, circRNAs are divided into four main types: exon circRNAs (ecRNAs)^5^, which mainly exist in the cytoplasm; intron circRNAs (ciRNAs)^6^, which are mainly located in the nucleus, circRNAs that are composed of exons and introns (EIciRNAs);^7^ and intergenic circRNAs^8^. In addition, there are tricRNAs, which are circRNAs derived from precursor tRNAs in the cytoplasm^9^. Most circRNAs are highly evolutionarily conserved among species, and many types of circRNAs have been proven to have higher expression levels than their corresponding linear RNAs, some by a factor of more than 10 (ref. ^10^). Because of their unique biological characteristics, circRNAs have become a focus of research into malignant tumors. Over time, four main functions of circRNAs have been discovered: (1) as miRNA “sponges”, they competitively bind to miRNAs and affect their expression; (2) they control gene expression as a transcriptional regulatory factor; (3) they bind to proteins to regulate a variety of biological processes; and (4) they participate in the process of protein translation. With the continuous progress of research on the biological function of circRNAs, the relationship between circRNAs and tumors has received increasing attention. CircRNA is considered an important molecular marker for the early diagnosis of tumors and a potential therapeutic target.

**Fig. 1:**
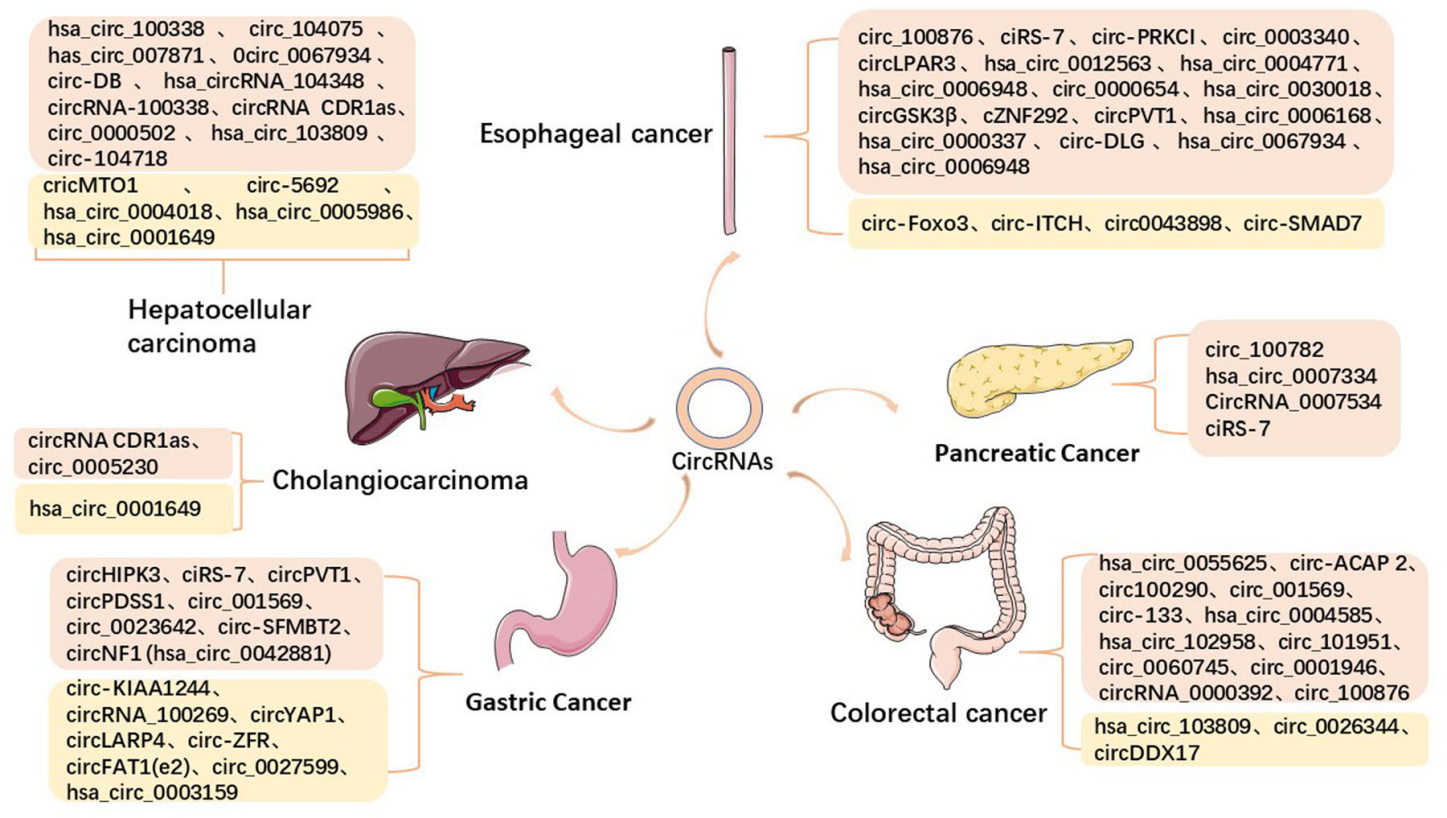
The expression of circRNA in digestive tract tumors. Some common disorders of circRNAs in digestive system neoplasms; the orange mark is the upregulated circRNAs, the golden mark is the downregulated circRNAs.

Malignant tumors of the digestive tract are an important part of the global tumor incidence and mortality, mainly including esophageal carcinoma, gastric cancer, pancreatic cancer, hepatocellular carcinoma, cholangiocarcinoma, colorectal cancer, etc. With the changes in people’s diet, the proportion of high-fat, high-protein, and low-fiber diets continues to increase, and the incidence of malignant tumors in the digestive tract is showing an increasing trend year by year. Although the improvement of medical standards has led to more and more treatments, the 5-year survival rate of advanced patients is still low due to the lack of effective early diagnosis^11,12^. Therefore, early diagnosis is essential for malignant tumors of the digestive tract. As the most common class of solid malignant tumors encountered in the clinic, malignant tumors of the digestive tract are a serious threat to human health. As established by an increasing number of studies, numerous circRNAs show abnormal expression in a variety of malignant tumors of the digestive tract and play an important biological role in the evolution and regulation of the disease (Fig. 1). Due to its structural stability and specificity, circRNAs play an important role in the early diagnosis of malignant tumors of the digestive tract, thereby achieving the purpose of early diagnosis and treatment and improving the survival rate of patients. In addition, due to the abnormal expression of circRNAs in cancer tissues, the circRNAs–miRNA–mRNA axis is used to regulate tumor progression, which affects the progression of the disease. Therefore, circRNAs can also be used as an effective target for cancer treatment, thereby improving the efficacy and prognosis of malignant tumors of the digestive tract.

## The formation process and characteristics of circRNA

**Fig. 2:**
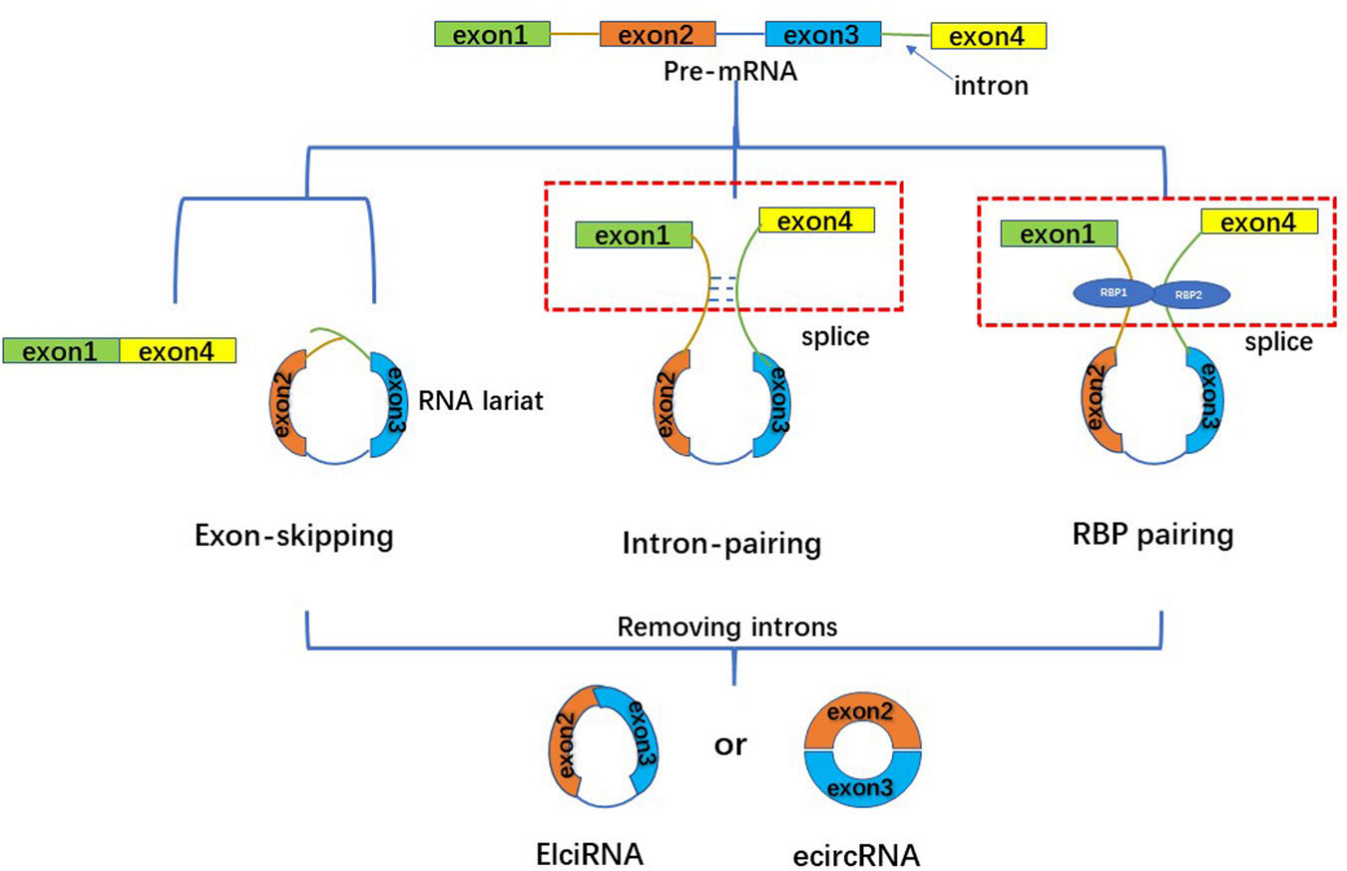
The formation process of ElciRNA and ecircRNA, including exon-skipping, intron-pairing, and RBP pairing. 1. Exon-skipping: the upstream exon and the downstream exon are covalently bonded to form an RNA lariat containing several exons and introns.The RNA lariat is spliced again, and the intron is removed to form ecircRNA or EicirRNA. 2.Intron-pairing: the two introns flanking circRNA have complementary structures. The splice sites are brought close by pairing and binding, and the introns are removed to form ecircRNA or EicirRNA. 3. RBP pairing: RBP binds upstream and downstream introns to form a bridge between the introns, and then remove the introns to produce ecircRNA or EicirRNA.

**Fig. 3:**
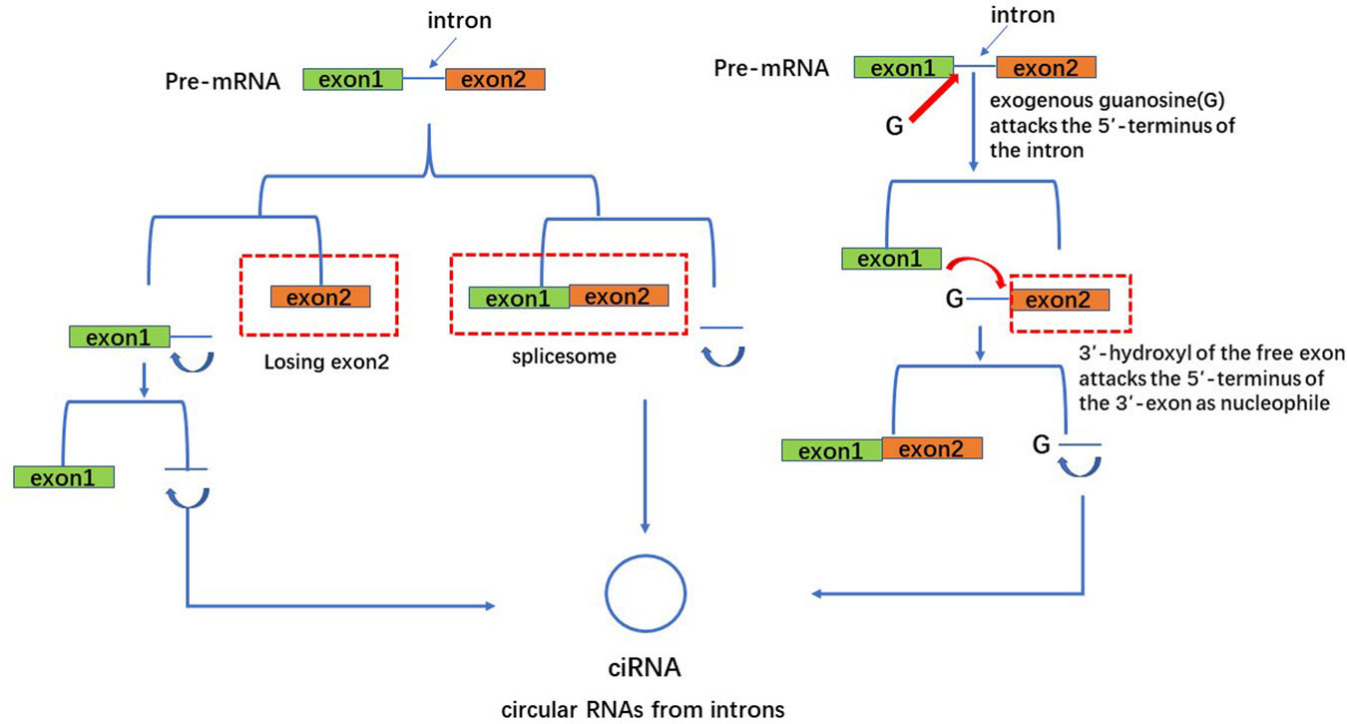
During the formation of ciRNA, exons are removed and introns are cyclized to form ciRNA. 1. A pre-mRNA is spliced by a spliceosome, producing an RNA lariat circularized with 2′,5′-phosphodiester. Finally, remove the 3′- tail of the RNA lariat and then form ciRNA. 2. The precursor RNA loses the 3′-exon. Then, the 2′-hydroxyl of the 3′-terminus attacks the 5′-terminus of the intron, producing a ciRNA. 3. Exogenous guanosine (G) attacks the 5′-terminus of the intron as nucleophile, causing the 5′-exon to be cut off. Then, the 3′- hydroxyl of the free exon acts as a nucleophile to attack the 5′-terminus of the 3′-exon, generating a linear intron. The linear introns then form a circularized RNA lariat, and finally ciRNA.

CircRNA is a ubiquitous class of endogenous noncoding RNA with a closed ring structure, consisting of 30 to 50 phosphodiester bonds. The formation of circRNA includes exon circularization and intron circularization (Figs. 2 and 3). There are three common processes of circRNA reverse splicing: exon skipping, intron pairing, and RNA-binding-protein-driven circularization. Exon skipping means that in the process of circRNA formation, the precursor mRNA reduces the spatial distance between exons by exon-skipping and forms a lariat structure in the form of covalent bonds; the exon lariat is spliced again, and the intron is removed to form circRNA. This process is known as “lariat-driven circularization”. On the other hand, intron pairing is a scenario in which the two introns flanking the circRNA have complementary structures, which bring the splice sites close together by pairing, forming a secondary structure and promoting reverse splicing. RNA-binding-protein-driven circularization relies on RNA-binding proteins to circularize the RNA^13^. The mechanisms of circRNA formation also include cyclization processes driven by tRNA splicing and rRNA splicing^14^. In addition, intron sequences, RNA polymerase II, and spliceosomes play an important role in the formation of circRNA^15^. To date, the formation process of circRNA has not been fully elucidated, and further research is needed.

## Biological function of circRNA

### CircRNAs as miRNA “sponges”

A miRNA is a small noncoding RNA with a length of approximately 22 nt. By binding to specific targets in the 3′-untranslated region (3′-UTR) of mRNA, it plays an important role in the regulation of posttranscriptional gene expression, reducing mRNA stability and inhibiting translation^16^. CircRNAs are not merely messengers between DNA and coding proteins; they can regulate the expression of target genes and ultimately affect the occurrence and development of disease by competitively binding with the corresponding miRNAs and affecting their activity. These circRNAs, also known as competitive endogenous RNAs (ceRNAs), are aptly compared to molecular “sponges”. CircRNA containing miRNA binding sites can inhibit the regulatory effect of miRNA on target gene mRNA, thus regulating the expression of target genes at the posttranscriptional level^17^. For example, circ_0055625, which is highly expressed in colon cancer, acts as a “sponge” for miR-106b to further promote the development of the disease^18^. On the other hand, the normal expression of hsa_circRNA_103809 shows an inhibitory effect on tumor growth, while the decreased expression of hsa_circRNA_103809 in colorectal cancer (CRC) eliminates its inhibitory effect and can promote the proliferation and migration of CRC cells through the miR532-3p/FOXO4 axis^19^. Therefore, the circRNA–miRNA–mRNA axis may play an important role in the regulation of tumor proliferation and migration. In addition, circRNA is structurally more stable than linear RNA; this stability, along with its tissue specificity, makes it a good biomarker.

### Regulation of gene expression as a transcriptional regulator

CircRNA can regulate gene expression at the transcriptional or posttranscriptional level by binding to miRNA or interacting with other molecules. In addition, recent studies have shown that some circRNAs can regulate parental gene expression. CircRNA can change the properties of RNA through base pairing; for example, CDR1as binds to CDR1 mRNA through complementary base pairing to form a “skeleton” structure, thus increasing the stability of CDR1 mRNA^20^. Some circRNAs located in the nucleus, such as EIciPAIP2 and EIciEIF3, have U1 snRNP binding sites, which can form RNA–RNA complexes and bind to RNA polymerase II (PolII) in the promoter region to regulate parental gene transcription^7^. Zhang et al. also found that ci-ankrd52 can directly regulate the expression of the ankrd52 gene by binding to the PolII, while knockdown of ci-ankrd52 can downregulate the expression of its parental gene^21^.

### Protein binding

Studies have shown that some circRNAs can bind to proteins at specific binding sites, thus affecting the biological functions of those proteins. Corm et al. found that circRNA can bind to Argonaute protein, thus inhibiting the translation function of the latter^22^. In addition, RNA-binding proteins not only participate in the formation of circRNA but also bind to circRNA to form a complex that further regulates gene expression. It has been found that the MBL protein can bind to the corresponding site of circRNA to form circMbl. In addition to inhibiting the further production of MBL protein, circMbl can block the production of new circMbl, indicating that circMbl is an autonomous regulator of MBL protein^23^. In addition, the binding of circ-PABPN1 from the PABPN1 locus to the RNA-binding protein human antigen R (HuR) inhibits its binding to PABPN1 mRNA, thus reducing cell proliferation^24^. In addition, Du et al. found that Circ-Foxo3 can bind to p21 and cyclin-dependent kinase 2 (CDK2) to form a circFoxo3–p21–CDK2 complex, which inhibits the function of CDK2 and induces cell cycle arrest^25^.

### Participation in the process of protein translation

Initially, circRNA was not considered to have a protein-coding function, but recent studies have gradually revealed that some circRNAs contain a highly conserved internal ribosome entry site (IRES) and can undergo translation. Legnini et al.^26^ found that circZNF609 expressed in mouse and human myoblasts with an open reading frame (ORF) across the reverse splicing site can be translated into protein in a splicing-dependent manner. In addition, circ-SHPRH and circ-FBXW7 and their encoded proteins are highly expressed in normal brains but downregulated in gliomas. These circRNAs have open reading frames that encode functional proteins with the help of internal ribosome entry sites, and their corresponding proteins SHPRH-146aa and FBXW7-185aa can inhibit the proliferation of glioma cells^27,28^.

## CircRNA and digestive system neoplasms

### Esophageal carcinoma (EC)

EC is one of the most common malignant tumors in the world. According to histological types, it can be divided into squamous cell carcinoma and adenocarcinoma. The high mortality rate of esophageal squamous cell carcinoma (ESCC) is mainly due to the low early diagnosis rate of the disease and about 50% of patients with tumor metastasis at the time of diagnosis. The 5-year survival rate of ESCC advanced patients is less than 20%, so early diagnosis and treatment can significantly improve the prognosis of ESCC patients, and the 10-year survival rate after surgery can reach 95%. Therefore, it is necessary to find molecular markers for the early diagnosis and prognosis of esophageal cancer.

#### Onco-circRNA in EC

The expression level of Circ_100876 in ESCC was found to be significantly increased; furthermore, its expression level was strongly correlated with the depth of invasion, lymph node metastasis, and vascular invasion of esophageal cancer cells, and the survival time of patients with high expression of Circ_100876 was significantly shortened. In addition, after the knockout of Circ_100876, the proliferation level of tumor cells decreased significantly (resulting in G2/M-phase cell cycle arrest and the occurrence of apoptosis in vitro), and the occurrence of cell metastasis, invasion, and epithelial mesenchymal transformation (EMT) was inhibited, which clearly indicated that Circ_100876 was closely related to the proliferation, metastasis, and invasion of esophageal cancer, such that it can be used as a marker to detect esophageal cancer. Indeed, Circ_100876 enables early identification and judgment of the occurrence and development of esophageal cancer^29^. In addition, Sang et al.^30^ found that the expression of ciRS-7 in ESCC is abnormally high, and the overexpression of ciRS7 can promote the proliferation, invasion, and migration of ESCC cells. In addition, there are binding sites between ciRS-7 and miR-876-5p as well as MAGEA family genes; binding at these sites can promote tumor proliferation, invasion, and migration. Other studies have shown that the upregulation of ciRS-7 can also eliminate the inhibitory effect of miR-7 on its downstream target gene HOXB13 and induce p65 phosphorylation, thus promoting the malignant biological behavior of ESCC^31^. CiRS-7 can also upregulate the phosphorylation of IKK-α by mediating miR-7/KLF4, thus promoting the malignant progression of ESCC^32^. It has been found that the circular RNA for protein kinase C (circ-PRKCI) is overexpressed in ESCC, and overexpressed circ-PRKCI can promote the proliferation and migration of tumor cells. In ESCC, miR-3680-3p is expressed at a low level and plays a role in inhibiting tumor growth, while circ-PRKCI can bind to miR-3680-3p and reverse its regulatory effect^33^.

In addition, some overexpressed circRNAs regulate biological processes in tumors through their own pathways; for example, circ_0003340 is overexpressed in ESCC and promotes tumor development through the miR-564/TPX2 pathway^34^. circLPAR3 upregulates MET gene expression by acting as a sponge for miR-198; activates the RAS/MAPK and PI3K/Akt pathways; and promotes the migration, invasion, and metastasis of ESCC cells in vivo and in vitro^35^. The expression of hsa_circ_0012563 in ESCC was significantly upregulated, while hsa_circ_0012563 knockout inhibited the XRCC1-mediated EMT pathway, which, in turn, inhibited cell migration and invasion^36^. Hsa_circ_0004771 is significantly upregulated in ESCC, and, by acting as a molecular sponge of miR-339-5p, it positively regulates CDC25A to promote the proliferation of ESCC^37^. Hsa_circ_0006948, which is overexpressed in ESCC tissue, can induce EMT and promote tumor progression by acting as a sponge for miR490-3p^38^. Circ_0000654 is the sponge for miR-149-5p, promoting the progression of esophageal squamous cell carcinoma by indirectly activating the IL-6/STAT3 signaling pathway^39^. Hsa_circ_0030018, as the sponge for miR-599, promotes the high expression of enabled homolog (ENAH) in EC cells and promotes tumor progression^40^. Through direct interaction with GSK3β, circGSK3β inhibits the activity of this enzyme, promotes the migration and invasion of ESCC cells, and provides a previously unrecognized mechanism for the occurrence and development of tumors^41^. In addition, circRNAcZNF292, circPVT1, hsa_circ_0006168, hsa_circ_0000337, circ-DLG1, and hsa_circ_0067934 are all highly expressed in ESCC and play important regulatory roles in the occurrence and development of esophageal cancer^42–47^.

#### Anti-oncogene circRNA in EC

Li et al.^48^ found that circ-ITCH is downregulated in ESCC, which exerts its antitumor effect by regulating the Wnt/β-catenin signal transduction pathway. In addition, the expression of circ-Foxo3 is low in ESCC, and the circ-Foxo3/miR-23a/PTEN pathway is the key pathway that inhibits the progression of ESCC^49^. Some circRNAs, such as circ0043898 and circ-SMAD7, show low expression in ESCC, while upregulation of their expression inhibits biological functions in esophageal cancer cells^50,51^.

### Gastric cancer (GC)

GC is the fifth most common malignant tumor in the world and the third leading cause of cancer deaths. Surgery is currently the only radical treatment, but many patients with gastric cancer still have a poor prognosis, mainly because early gastric cancer is difficult to diagnose. Therefore, finding new molecular markers and effective therapeutic targets for early diagnosis is the current research focus. In recent years, great progress has been made in exploring the relationship between circRNA and GC. After a large number of studies, a variety of circRNAs have been found to be abnormally expressed in GC tissues, and the biological behavior of tumor cells can be regulated through the circRNA–miRNA–mRNA axis.

#### Onco-circRNA in GC

In general, circRNA and the corresponding miRNA have a negative regulatory relationship when participating in the process of GC cell proliferation. For example, circHIPK3 is highly expressed in GC, while miR-124 and miR-29b are continuously downregulated in GC, and circHIPK3 negatively regulates miR-124/miR-29b. The circHIPK3-miR-124/miR-29b axis can regulate multiple genes, thus further regulating the development of GC^52^. In addition, it has been found that ciRS-7 plays a carcinogenic role in GC cells by antagonizing the miR-7-mediated PTEN/PI3K/AKT pathway. Overexpression of ciRS-7 can counteract the inhibitory effect of miR-7 on the migration of GC cells and reduce the apoptosis induced by miR-7 (ref. ^53^). Chen et al.^54^ found that CircPVT1 is highly expressed in GC tissues, promoting the progression of GC by acting as a sponge for miR-125. In addition, circPDSS1, circRNA_001569, circRNA_0023642, circ-SFMBT2, and circNF1 (hsa_circ_0042881) are also highly expressed in GC tissues, regulating the occurrence and development of GC through different ways^55^. By acting as a sponge for miR-186-5p, circPDSS1 inhibits the biological function of miR-186-5p, further inhibits the downstream target NEK2, and then regulates cell proliferation and apoptosis, thus promoting the development of GC. On the other hand, circRNA_001569 interacts with miR-145, inhibits the biological activity of this miRNA, and further inhibits the expression of NR4A2, the downstream target of miR-145; thus, it significantly improves the vitality of GC cells, promotes proliferation and reduces apoptosis. CircRNA_0023642 is a metastasis-activating factor. It promotes cell proliferation, metastasis, and invasion by regulating the EMT signaling pathway^56^. Circ-SFMBT2 is highly expressed in GC tissues and cells, as well as the plasma of GC patients, and is positively correlated with TNM stage. It can target miR-182-5p and further regulate the expression of the oncogene CREB1, which promotes cell proliferation. On the other hand, circNF1 (hsa_circ_0042881) can regulate biological processes in GC by binding to miR-16 and then downregulating the expression of MAP7 and AKT3 mRNA.

#### Anti-oncogene circRNA in GC

In addition, there are some low-expression circRNAs in GC tissues that also play important roles in regulating the occurrence and development of GC. For example, circ-KIAA1244 (ref. ^57^), hsa_circ_0003159 (ref. ^58^), circRNA_100269, circYAP1, CircLARP4, circ-ZFR, circFAT1 (e2), and circ_0027599 (ref. ^55^) show low expression in GC, but their overexpression can inhibit the occurrence and progression of tumors. Therefore, these disorders of circRNA expression play important regulatory roles in the occurrence and development of GC and provide new ideas for the early diagnosis and comprehensive treatment of GC.

### Pancreatic cancer (PC)

PC is one of the digestive tract tumors with high malignancy and poor prognosis. In recent years, its incidence and mortality have been increasing year by year. Due to the difficulty in diagnosing early pancreatic cancer, more than 90% of patients are already in the middle and advanced stages when they are diagnosed with pancreatic cancer, and they have lost the best time for treatment. Their 5-year survival rate is less than 5%. Therefore, early diagnosis and early treatment are essential for pancreatic cancer. In recent years, it has been confirmed that circRNA plays an important regulatory role in the progression of pancreatic ductal adenocarcinoma (PDAC). Its abnormal expression affects the proliferation of tumor cells and the development of PDAC. Studies have shown that the expression of circRNA_100782 in PDAC tissue is significantly upregulated and plays a positive role in regulating the proliferation of tumor cells. MiR-124 is a negative regulator of cell proliferation. Chen et al. found that circRNA_100782 inhibits the biological activity of miR-124 and further activates its downstream targets, interleukin-6 receptor (IL6R) and signal transducer and activator of transcription 3 (STAT3), by acting as a sponge for miR-124, thus enabling cell proliferation to be promoted. Experiments have shown that downregulating the expression of circRNA_100782 inhibits the proliferation and colony formation of BxPC3 PDAC cells, which further illustrates the role of circRNA_100782 in promoting cell proliferation in PDAC^59^. In addition, Yang et al.^60^ found that hsa_circRNA_0007334 was significantly upregulated in PDAC tissues, which may enhance the expression of matrix metallopeptidase 7 (MMP7) and collagen type I alpha 1 (COL1A1) by competitive adsorption of hsa-miR-144-3p and hsa-miR-577, thus regulating biological processes in PDAC. In addition, circRNA_0007534 and ciRS-7 are also highly expressed in PDAC tissues; circRNA_0007534 regulates miR-625 and miR-892b, increasing the carcinogenicity of PDAC^61^, while ciRS-7 targets miR-7 and regulates the EGFR/STAT3 signal pathway, thus playing a carcinogenic role^62^.

### Hepatocellular carcinoma (HC)

HC is the sixth most common malignant tumor in the world, and the mortality rate ranks fourth. Due to the insidious disease, most patients are already at an advanced stage when HC is diagnosed, and they are prone to recurrence and metastasis after surgery, and the 5-year survival rate of patients is low. At present, serum alpha-fetoprotein (APF) combined with ultrasound is the most extensive screening for early HC. However, the accuracy of ultrasound examination mainly depends on the sensitivity of the instrument and the level of the operator, and it is not easy to diagnose small malignant nodules. AFP has the phenomenon of low sensitivity and high false positive rate, and it is not ideal for screening early HC. Therefore, looking for new potential biomarkers has important clinical significance for improving the early diagnosis of HC. At present, a variety of circRNAs have been found to play important roles in the progression of HC. They combine with specific miRNAs and affect the expression of downstream targets to promote the proliferation and metastasis of tumor cells. Further studies have established circRNA as a potential biomarker for the early clinical diagnosis and prognosis evaluation of HC.

#### Onco-circRNA in HC

Huang et al. found that hsa_circ_100338 is upregulated and is an important gene regulator in HC. It can act on miR-141-3p and regulate the metastasis of HC through the circRNA_100338/miR141-3p/MTSS1 axis. The expression level of hsa_circ_100338 in HC is closely related to tumor metastasis and the rate of patient survival. In addition, the study found that an increase in hsa_circ_100338 can also activate the mTOR signaling pathway in HC through the circRNA_100338/miR141-3p/RHEB axis and is related to poor prognosis in patients with hepatitis B-related HC^63,64^. In addition, Zhang et al.^65^ found that circ_104075 is abundant in liver cancer tumor tissues, cells, and patient serum and can act as a sponge for miR-582-3p to upregulate the expression of the downstream target YAP. In addition, the more serious the condition of patients with HC, the higher their serum levels of circ_104075. An analysis of serum circ_104075 levels before and after operation showed that the level decreased significantly after operation. Therefore, circ_104075 is a promising serum marker for the diagnosis of HC. In addition, hsa_circ_0078710 was confirmed to be overexpressed in HepG2 and SMMC-7721 cell lines and upregulated the expression of HDAC and CDK2 by acting as a sponge for miR-31; this circRNA was also found to mediate the expression of cell cycle components (cyclin A, cyclin D1, CDK4) and p21, which further induced cell cycle progression and significantly promoted cell proliferation, migration, invasion, and growth^66^. Circ_0067934 is also upregulated in HC tissues and cell lines, while knockout of the circ_0067934 gene can inhibit proliferation, migration, invasion, and apoptosis in Hep3B and Huh7 cells. Circ_0067934 can directly inhibit the expression of miR-1324 and then regulate mRNA-FZD5 to further downregulate the Wnt/β-catenin signaling pathway to promote the proliferation, migration, and invasion of HC cells^67^. One of the more special circRNAs is the one secreted by adipocytes, namely, circ-DB. It regulates the growth of hepatoma cells through the circ-DB/miR-34a/USP7/cyclin A2 pathway. In HCC patients with high body fat percentages, the expression of circ-DB is upregulated, and circ-DB downregulates the expression of miR-34a by acting as a miRNA sponge, thus activating USP7, which can promote tumor growth and metastasis by reducing the ubiquitination of Cyclin A2 and many other proteins^68^. Huang et al.^69^ indicated that circular RNA hsa_circRNA_104348 might function as a competing endogenous RNA (ceRNA) to promotes HCC progression by targeting miR-187-3p/RTKN2 axis and activating Wnt/β-catenin pathway. Another study indicated that metastatic ability of HCC cells could be enhanced by transferring exosomal circRNA-100338 to recipient HUVECs, which could affect proangiogenic activity by regulating angiogenesis^70^. CircRNAs such as circRNA-Cdr1as^71^, circRNA_0000502, and hsa_circRNA_103809 are highly expressed in HCC tissues. All of them can regulate biological functions in HCC and promote the progression of HCC through different targeted pathways^72,73^. In addition, recent studies have found that some circRNA have a certain effect on the resistance of sorafenib. Xu et al. found that CircRNA-SORE plays an important role in it. It can mediate sorafenib resistance in hepatocellular carcinoma by stabilizing YBX1, and N-methylated CircRNA-SORE can also maintain sorafenib resistance through β-catenin signaling^74,75^.

#### Anti-oncogene circRNA in HC

Other circRNAs are different; they show low expression in HC tissues and serve the biological function of inhibiting tumor occurrence and progression. For example, circMTO1, which is significantly downregulated in HC, can be used as a sponge for miR-9 to promote the expression of P21 and to inhibit the proliferation and invasion of HC^76^. Additionally, Wang et al. found that circMTO1 can also exert its inhibitory effect through the miR-9-5p/NOX4 axis^77^. In addition, circRNA-5692 is expressed at low levels in HCC. It interacts with miR-328-5p and targets mRNA-DAB2IP to inhibit the progression of HCC^78^. In addition, the hsa_circ_0004018/miR30e-5p/miR-626-MYC axis^79^, the hsa_circ_0005986/miR-129-5p/Notch1 axis^80^, and hsa_circ_0001649/miR-127-5p/miR-612/miR-4688 axis^81^ all play important roles in regulating tumor progression.

### Cholangiocarcinoma (CCA)

CCA refers to a malignant tumor derived from the bile duct epithelium. According to different anatomical locations, CCA is divided into intrahepatic cholangiocarcinoma, hilar cholangiocarcinoma, and distal cholangiocarcinoma. Because it has no outstanding clinical manifestations at the time of onset, it is often in the terminal stage at the time of diagnosis, and faces problems such as insufficient treatment, poor prognosis, and low survival rate. The overall 5-year survival rate after surgery is less than 5%. Recent studies have shown that CircRNA also plays an important role in the occurrence and development of CCA. Studies have shown that the expression of circRNACDR1as is significantly higher in CCA tumor tissues than in paracancerous tissues, and the overexpression of CDR1as is closely related to the TNM stage, lymphatic invasion, and postoperative recurrence of the tumor. The overall survival rate of CCA patients with high expression of CDR1as is significantly lower than that of CCA patients with low expression of CDR1as. Therefore, the expression level of CDR1as can be used as an independent biomarker to predict the prognosis of CCA, achieving good sensitivity and specificity^82^. Several studies, including one by Li, have shown that CDR1as can bind to miR-641 and accelerate its degradation, then activate the AKT3/mTOR pathway and promote the proliferation, migration, and invasion of CCA cells^83^. In addition, Xu et al.^84^ found that circ_0005230 is highly expressed in CCA, playing a carcinogenic role by acting as a sponge for miR-1238 and miR-1299, and is positively correlated with clinical severity. The other circRNA, hsa_circ_0001649, is scarce in CCA tissues and cells, while high levels of hsa_circ_0001649 can inhibit the proliferation, migration, and invasion of CCA cells and induce cell apoptosis to exert a tumor suppressor effect^85^.

### Colorectal cancer (CRC)

CRC is a common type of malignant tumor of the digestive tract. Approximately 1.36 million people worldwide suffer from CRC, which accounts for 10.2% of all cancer populations. Its mortality rate ranks second among malignant tumors. The treatment of CRC is mainly surgery, combined with radiotherapy and chemotherapy, targeted therapy, and immunotherapy. The 5-year and 10-year relative survival rates of CRC patients are 65% and 58%, respectively. If cancer in situ is diagnosed, the 5-year survival rate of CRC patients can reach 90%; when tumor cells spread and metastasize, the 5-year survival rate of CRC patients drops to about 11.7%. Therefore, early diagnosis and early treatment of CRC are essential. In CRC, circRNA plays an important role in the regulation of tumor proliferation, metastasis, and invasiveness. To date, a variety of abnormal expression of circRNA has been found in the course of CRC; such abnormalities can play a key role in regulating the occurrence and progression of CRC through different biological mechanisms.

#### Onco-circRNA in CRC

Studies have shown that the hsa_circ_0055625/miR-106b/ITGB8 pathway may play an important regulatory role in the proliferation, migration, and invasion of CRC^18^. The expression of circ_0055625 is significantly upregulated in CRC and is related to tumor size, pathological stage, histological differentiation, and lymph node metastasis; that is, higher expression is associated with larger CRC size, higher TNM stages, and higher histological grades. In addition, the circRNA-ACAP2/hsa-miR-21-5p/Tiam1 regulatory pathway can affect the proliferation, migration, and invasion of colon cancer SW480 cells. In CRC tissues and SW480 cells, the expression of circRNA-ACAP2 and Tiam1 is increased, while the expression of miR-21-5p is decreased, indicating that circRNA-ACAP2 and Tiam1 may promote the growth of tumor cells, while miR-21-5p acts as an inhibitor of tumor growth^86^. Guan et al.^87^ found that the circRNA100290/miR516b/FZD4/Wnt/β-catenin pathway is also closely related to the progression of CRC. The expression of circRNA100290 is upregulated in CRC tissues and cells. Silencing circRNA100290 can significantly inhibit the proliferation, migration, and invasion of CRC cells and promote apoptosis. The mechanism may be that circRNA100290 binds to miR-516b, which affects the activity of its downstream target FZD4, thus activating the Wnt/β-catenin signaling pathway to affect the proliferation, migration, and invasiveness of tumor cells. The expression of circ_001569 in CRC tissues is also significantly upregulated and can significantly promote the proliferation and invasion of tumor cells. Circ_001569, as a miRNA sponge, can directly inhibit the expression of miR-145 and then upregulate the expression of its downstream targets E2F5, BAG4, and FMNL2, which gives it a tumor-promoting role in CRC cells. Among these molecules, miR-145 is negatively correlated with circ_001569, E2F5, BAG4, and FMNL2 (ref. ^88^). Yang et al. found that Hypoxia-derived exosomal circ-133 transported into normaxic cancer cells and promoted cell migration via miR-133a/GEF-H1/RhoA axis. This reveals a potential mechanism for that the intra-tumor heterogeneity of oxygen promote cancer progression^89^. In addition, hsa_circ_0004585, hsa_circRNA_102958, circRNA_101951, circ_0060745, circ_0001946, circRNA_0000392, and circRNA_100876 are highly expressed in CRC and regulate tumor growth through their own pathways.

#### Anti-oncogene circRNA in CRC

Other circRNAs play inhibitory roles in CRC: hsa_circRNA_103809 is expressed at low levels in CRC, and as a tumor suppressor gene, it regulates tumor cell proliferation and migration through the miR-532-3p/FOXO4 axis;^19^ circRNA_0026344 also acts as a tumor suppressor gene to affect the occurrence and development of tumors. The expression of circRNA_0026344 in CRC is significantly downregulated, and it functions as a sponge for miR-21/miR-31. The downregulation of circRNA_0026344 levels will lead to an increase in CRC progression and lymph node metastasis; therefore, low expression of circRNA_0026344 may predict a poor prognosis in CRC patients^90^. Li et al. found that Circ-DDX17 is significantly downregulated in CRC tissue and plays a tumor-suppressing role; thus, it can be used as a potential biomarker and therapeutic target for CRC^91^.

## Issues and prospects

To date, circRNAs and miRNAs have been proven to regulate the occurrence and progression of tumors through a variety of small molecules and signaling pathways; accordingly, they have become new targets for tumor therapy. As a miRNA sponge, circRNA specifically binds to miRNAs and regulates gene expression, which provides a new means for further understanding the language of RNA and its role in human disease signaling pathways. As mentioned above, circRNA shows great potential as a biomarker and treatment target for malignant tumors of the digestive tract. Because of its specific expression and ring structure, it can be used as a biological marker for the prediction of human diseases, thus improving the accuracy and specificity of diagnosis and treatment. In addition, due to the abnormal expression of circRNA in cancer tissues, circRNA can also be used as a therapeutic target to improve the prognosis of malignant digestive tract tumors and the efficacy of their treatment (Table 1). However, many aspects of circRNA are still unknown, and there are some problems to be solved, such as the following: (1) How is circRNA normally degraded in cells? It is very important to find a suitable degradation pathway for overexpressed circRNAs that promote cancer. For example, hsa_circ_100338 is upregulated and is an important gene regulator in HC. It regulates the metastasis of HC cells through the circRNA_100338/miR141-3p/MTSS1 axis. Accurately degrading circRNA and cutting off the pathway from the source is an important and effective means to inhibit cancer. (2) The tissue contains a large number of circRNAs that have different functions. Great progress has been made in the study of single circRNAs. However, multiple circRNAs can be dysregulated in the same cancer tissue, and it is often not known whether their pathways are related to each other, that is, whether they have a common target. For example, in gastric cancer, a variety of circRNAs, such as circHIPK3, ciRS-7, and circPVT1, are upregulated to promote the occurrence and development of cancer, while circ-KIAA1244, circRNA_100269, and circ-YAP1, which play various roles in inhibiting cancer, are downregulated in gastric cancer. There are various circRNA expression disorders with different mechanisms; which of these circRNA pathways play stronger and more obvious roles is a question that remains to be answered, and whether there is a common pathway and common regulatory factors also needs to be further explored. Are there any synergistic or antagonistic relationships among these different pathways? These problems need to be investigated. (3) The combination of circRNA and miRNA plays an important role in circRNA pathways, such as the circ_0003340/miR-564/TPX2 pathway and hsa_circ_0004771/miR-339-5p/CDC25A in esophageal cancer. In theory, blocking the binding sites of selected circRNAs and miRNAs might also effectively inhibit the occurrence and development of cancer. Therefore, research is needed to verify whether their effects can be accurately inhibited by blocking their binding sites. In addition, circRNA is still a long way from practical use as a biomarker. Testing for circRNA in tissue is more expensive and can cause some pain to patients; additionally, the reliability of diagnosis with circRNA remains to be confirmed.

**Table 1:** Some important circRNAs in the digestive system.

Disease	Dysregulation	CircRNA	miRNA	Biological functions in cancer
Esophageal cancer	Upregulated	circ_100876	-	Promotion
		ciRS-7	miR-876-5p、miR-7	Promotion
		circ-PRKCI	miR-3680-3p	Promotion
		circ_0003340	miR-564	Promotion
		circLPAR3	miR-198	Promotion
		hsa_circ_0012563	-	Promotion
		hsa_circ_0004771	miR-339-5p	Promotion
		hsa_circ_0006948	miR-490-3p	Promotion
		circ_0000654	miR-149-5p	Promotion
		hsa_circ_0030018	miR-599	Promotion
		circGSK3β	-	Promotion
		cZNF292	miR-206	Promotion
		circPVT1	miR-4663	Promotion
		hsa_circ_0006168	miR-100	Promotion
		hsa_circ_0000337	miR-670-5p	Promotion
		circ-DLG1	-	Promotion
		hsa_circ_0067934	-	Promotion
		hsa_circ_0006948	-	Promotion
	Downregulated	circ-Foxo3	miR-23a	Inhibition
		circ-ITCH	miR-7、miR-17、miR-214	Inhibition
		circ0043898	-	Inhibition
		circ-SMAD7	-	Inhibition
Gastric cancer	Upregulated	circHIPK3	miR-124、miR-29b	Promotion
		ciRS-7	miR-7	Promotion
		circPVT1	miR-125	Promotion
		circPDSS1	miR-186-5p	Promotion
		circ_001569	miR-145	Promotion
		circ_0023642	-	Promotion
		circ-SFMBT2	miR-182-5p	Promotion
		circNF1 (hsa_circ_0042881)	miR-16	Promotion
	Downregulated	circ-KIAA1244	-	Inhibition
		circRNA_100269	miR-630	Inhibition
		circYAP1	miR-367-5p	Inhibition
		circLARP4	miR-424-5p	Inhibition
		circ-ZFR	miR-130a、miR-107	Inhibition
		circFAT1(e2)	miR-548g	Inhibition
		circ_0027599	miR-101	Inhibition
		hsa_circ_0003159	miR-223-3p	Inhibition
Pancreatic cancer	Upregulated	circ_100782	miR-124	Promotion
		hsa_circ_0007334 circRNA_0007534 ciRS-7	hsa-miR-144-3p、 hsa-miR-577 miR-625、miR-892b miR-7	Promotion Promotion Promotion
Hepatocellular carcinoma	Upregulated	hsa_circ_100338	miR141-3p	Promotion
		circ_104075	miR-582-3p	Promotion
		has_circ_0078710	miR-31	Promotion
		circ_0067934	miR-1324	Promotion
		circ-DB hsa_circRNA_104348 circRNA-100338	miR-34a miR-187-3p-	Promotion Promotion Promotion
		circRNA Cdr1as	miR-1270	Promotion
		circ_0000502	miR-124	Promotion
		hsa_circ_103809	miR-377-3p	Promotion
		circ-104718	miR-218-5p	Promotion
	Downregulated	cric MTO1	miR-9、miR-9-5p	Inhibition
		circ-5692	miR-328-5p	Inhibition
		hsa_circ_0004018	miR-30e-5p、miR-626	Inhibition
		hsa_circ_0005986	miR-129-5p	Inhibition
		hsa_circ_0001649	-	Inhibition
Cholangiocarcinoma	Upregulated	circRNA CDR1as	miR-641	Promotion
		circ_0005230	miR-1238、miR-1299	Promotion
	Downregulated	hsa_circ_0001649	-	Inhibition
Colorectal cancer	Upregulated	hsa_circ_0055625	miR-106b	Promotion
		circ-ACAP 2	miR-21-5p	Promotion
		circ100290	miR-516b	Promotion
		circ_001569 circ-133	miR-145 miR-133a	Promotion Promotion
		hsa_circ_0004585	-	Promotion
		hsa_circ_102958	miR-585	Promotion
		circ_101951	-	Promotion
		circ_0060745	miR-4736	Promotion
		circ_0001946 circRNA_0000392	miR-135a-5p miR-193a-5p	Promotion Promotion
		circ_100876	miR-516b	Promotion
	Downregulated	hsa_circ_103809	miR-532-3p	Inhibition
		circ_0026344	miR-21、miR-31	Inhibition
		circDDX17	-	Inhibition

*CircRNA* circular RNA, *miR* microRNA.

## Conclusion

In summary, circRNA plays an important regulatory role in the occurrence and development of digestive system neoplasms. It not only provides a new means for early treatment and prognosis judgment but also serves as a new target for tumor treatment. To date, circRNA has not been studied in depth, and there is a clinically significant question of how to further explore the mechanisms and pathological role of circRNA and to put forward an effective and highly targeted diagnosis and treatment plan; future circRNA research will be directed toward these goals.

## References

[1] Memczak S (2013). Circular RNAs are a large class of animal RNAs with regulatory potency. Nature.

[2] Zhang Z, Yang T, Xiao J (2018). Circular RNAs: promising biomarkers for human diseases. EBIO Med..

[3] Salzman J, Gawad C, Wang PL, Lacayo N, Brown PO (2012). Circular RNAs are the predominant transcript isoform from hundreds of human genes in diverse cell types. PLoS One.

[4] Jeck WR (2013). Circular RNAs are abundant, conserved, and associated with ALU repeats. RNA.

[5] Zhang XO (2014). Complementary sequence-mediated exon circularization. Cell.

[6] Talhouarne GJS, Gall JG (2018). Lariat intronic RNAs in the cytoplasm of vertebrate cells. Proc. Natl Acad. Sci. USA.

[7] Li Z (2015). Exon–intron circular RNAs regulate transcription in the nucleus. Nat. Struct. Mol. Biol..

[8] Geng Y, Jiang J, Wu C (2018). Function and clinical significance of circRNAs in solid tumors. J. Hematol. Oncol..

[9] Lu Z (2015). Metazoan tRNA introns generate stable circular RNAs in vivo. RNA.

[10] Wang Y (2019). Exosomal circRNAs: biogenesis, effect and application in human diseases. Mol. Cancer.

[11] Miller KD (2019). Cancer treatment and survivorship statistics. CA Cancer J. Clin..

[12] Siegel RL, Miller KD, Jemal A (2020). Cancer statistics, 2020. CA Cancer J. Clin..

[13] Meng S (2017). CircRNA: functions and properties of a novel potential biomarker for cancer. Mol. Cancer.

[14] Zhang Y (2016). The biogenesis of nascent circular RNAs. Cell Rep..

[15] Ashwal-Fluss R (2014). circRNA biogenesis competes with pre-mRNA splicing. Mol. Cell.

[16] Bartel DP (2009). MicroRNAs: target recognition and regulatory functions. Cell.

[17] Salmena L, Poliseno L, Tay Y, Kats L, Pandolfi PP (2011). A ceRNA hypothesis: the Rosetta Stone of a hidden RNA language?. Cell.

[18] Zhang J, Liu H, Zhao P, Zhou H, Mao T (2019). Has_circ_0055625 from circRNA profile increases colon cancer cell growth by sponging miR-106b-5p. J. Cell Biochem..

[19] Bian L (2018). Hsa_circRNA_103809 regulated the cell proliferation and migration in colorectal cancer via miR-532-3p / FOXO4 axis. Biochem. Biophys. Res. Commun..

[20] Hansen TB (2013). Natural RNA circles function as efficient microRNA sponges. Nature.

[21] Zhang Y (2013). Circular intronic long noncoding RNAs. Mol. Cell.

[22] Conn SJ (2015). The RNA binding protein quaking regulates formation of circRNAs. Cell.

[23] Ebbesen KK, Kjems J, Hansen TB (2016). Circular RNAs: identification, biogenesis and function. Biochim. Biophys. Acta.

[24] Abdelmohsen K (2017). Identification of HuR target circular RNAs uncovers suppression of PABPN1 translation by CircPABPN1. RNA Biol..

[25] Du WW (2016). Foxo3 circular RNA retards cell cycle progression via forming ternary complexes with p21 and CDK2. Nucleic Acids Res..

[26] Legnini I (2017). Circ-ZNF609 is a circular RNA that can be translated and functions in myogenesis. Mol. Cell.

[27] Zhang M (2018). A novel protein encoded by the circular form of the SHPRH gene suppresses glioma tumorigenesis. Oncogene.

[28] Yang Y (2018). Novel role of FBXW7 circular RNA in repressing glioma tumorigenesis. J. Natl Cancer Inst..

[29] Cao S, Chen G, Yan L, Li L, Huang X (2018). Contribution of dysregulated circRNA_100876 to proliferation and metastasis of esophageal squamous cell carcinoma. Onco. Targets Ther..

[30] Sang M (2018). Circular RNA ciRS-7 accelerates ESCC progression through acting as a miR-876-5p sponge to enhance MAGE-A family expression. Cancer Lett..

[31] Li RC (2018). CiRS-7 promotes growth and metastasis of esophageal squamous cell carcinoma via regulation of miR-7/HOXB13. Cell Death Dis..

[32] Huang H (2019). Circular RNA ciRS-7 triggers the migration and invasion of esophageal squamous cell carcinoma via miR-7 /KLF4 and NF-κB signals. Cancer Biol. Ther..

[33] Shi N (2019). Circular RNA circ-PRKCI functions as a competitive endogenous RNA to regulate AKT3 expression by sponging miR-3680-3p in esophageal squamous cell carcinoma. J. Cell Biochem..

[34] Hou Y, Liu H, Pan W (2020). Knockdown of circ_0003340 induces cell apoptosis, inhibits invasion and proliferation through miR-564/TPX2 in esophageal cancer cells. Exp. Cell Res..

[35] Shi Y (2020). Circular RNA LPAR3 sponges miR-198 to facilitate esophageal cancer migration, invasion and metastasis. Cancer Sci..

[36] Zhang Z, Li X, Xiong F, Ren Z, Han Y (2020). Hsa_circ_0012563 promotes migration and invasion of esophageal squamous cell carcinoma by regulating XRCC1/EMT pathway. J. Clin. Lab. Anal..

[37] Huang E (2020). CircRNA hsa_circ_0004771 promotes esophageal squamous cell cancer progression via miR-339-5p/CDC25A axis. Epigenomics.

[38] Pan Z (2020). Hsa_circ_0006948 enhances cancer progression and epithelial-mesenchymal transition through the miR-490-3p/HMGA2 axis in esophageal squamous cell carcinoma. Aging.

[39] Xu Z (2020). Circular RNA hsa_circ_0000654 promotes esophageal squamous cell carcinoma progression by regulating the miR-149-5p/IL-6/STAT3 pathway. IUBMB Life.

[40] Wang C (2019). Circular RNA hsa_circ_0030018 acts as a sponge of miR-599 to aggravate esophageal carcinoma progression by regulating ENAH expression. J. Cell Biochem..

[41] Hu X (2019). circGSK3β promotes metastasis in esophageal squamous cell carcinoma by augmenting β-catenin signaling. Mol. Cancer.

[42] Liu Z (2020). Silence of cZNF292 suppresses the growth, migration, and invasion of human esophageal cancer Eca-109 cells via upregulating miR-206. J. Cell Biochem..

[43] Zhong R, Chen Z, Mo T, Li Z, Zhang P (2019). Potential role of circPVT1 as a proliferative factor and treatment target in esophageal carcinoma. Cancer Cell Int..

[44] Shi Y (2019). hsa_circ_0006168 sponges miR-100 and regulates mTOR to promote the proliferation, migration and invasion of esophageal squamous cell carcinoma. Biomed. Pharmacother..

[45] Song H (2019). Upregulated circ RNA hsa_circ_0000337 promotes cell proliferation, migration, and invasion of esophageal squamous cell carcinoma. Cancer Manag. Res..

[46] Rong J (2018). Circ-DLG1 promotes the proliferation of esophageal squamous cell carcinoma. Onco. Targets Ther..

[47] Xia W (2016). Circular RNA has_circ_0067934 is upregulated in esophageal squamous cell carcinoma and promoted proliferation. Sci. Rep..

[48] Li F (2015). Circular RNA ITCH has inhibitory effect on ESCC by suppressing the Wnt/β-catenin pathway. Oncotarget.

[49] Xing Y (2020). Circular RNA circ-Foxo3 inhibits esophageal squamous cell cancer progression via the miR-23a/PTEN axis. J. Cell Biochem..

[50] Wang W (2018). Circ0043898 acts as a tumor inhibitor and performs regulatory effect on the inhibition of esophageal carcinoma. Cancer Biol. Ther..

[51] Zhang Y (2019). Upregulation of circ-SMAD7 inhibits tumor proliferation and migration in esophageal squamous cell carcinoma. Biomed. Pharmacother..

[52] Cheng J (2018). Regulatory network of circRNA–miRNA–mRNA contributes to the histological classification and disease progression in gastric cancer. J. Transl. Med..

[53] Pan H (2018). Overexpression of circular RNA ciRS-7 abrogates the tumor suppressive effect of miR-7 on gastric cancer via PTEN/PI3K/AKT signaling pathway. J. Cell Biochem..

[54] Chen J (2017). Circular RNA profile identifies circPVT1 as a proliferative factor and prognostic marker in gastric cancer. Cancer Lett..

[55] Li R (2020). CircRNA: a rising star in gastric cancer. Cell Mol. Life Sci..

[56] Zhou LH (2018). CircRNA_0023642 promotes migration and invasion of gastric cancer cells by regulating EMT. Eur. Rev. Med. Pharm. Sci..

[57] Tang W (2018). CircRNA microarray profiling identifies a novel circulating biomarker for detection of gastric cancer. Mol. Cancer.

[58] Wang J (2020). Hsa_circ_0003159 inhibits gastric cancer progression by regulating miR-223-3p/NDRG1 axis. Cancer Cell Int..

[59] Chen G, Shi Y, Zhang Y, Sun J (2017). CircRNA_100782 regulates pancreatic carcinoma proliferation through the IL6-STAT3 pathway. Onco Targets Ther..

[60] Yang J., et al. Circular RNA hsa_circRNA_0007334 is predicted to promote MMP7 and COL1A1 expression by functioning as a miRNA sponge in pancreatic ductal adenocarcinoma. *J. Oncol.* **2019**, 7630894 (2019). 10.1155/2019/7630894 PMC6681607 31428151

[61] Hao L (2019). Upregulated circular RNA circ_0007534 indicates an unfavorable prognosis in pancreatic ductal adenocarcinoma and regulates cell proliferation, apoptosis, and invasion by sponging miR-625 and miR-892b. J. Cell Biochem..

[62] Liu L (2019). Circular RNA ciRS-7 promotes the proliferation and metastasis of pancreatic cancer by regulating miR-7-mediated EGFR/STAT3 signaling pathway. HBPD INT.

[63] Huang XY (2017). Comprehensive circular RNA profiling reveals the regulatory role of the circRNA-100338/miR-141-3p pathway in hepatitis B-related hepatocellular carcinoma. Sci. Rep..

[64] Huang XY (2019). CircRNA-100338 is associated with mTOR signaling pathway and poor prognosis in hepatocellular carcinoma. Front Oncol..

[65] Zhang X (2018). circRNA_104075 stimulates YAP-dependent tumorigenesis through the regulation of HNF4a and may serve as a diagnostic marker in hepatocellular carcinoma. Cell Death Dis..

[66] Xie B (2019). CircRNA has_circ_0078710 acts as the sponge of microRNA-31 involved in hepatocellular carcinoma progression. Gene.

[67] Zhu Q (2018). CircRNA circ_0067934 promotes tumor growth and metastasis in hepatocellular carcinoma through regulation of miR-1324/FZD5/Wnt/β-catenin axis. Biochem. Biophys. Res. Commun..

[68] Zhang H (2019). Exosome circRNA secreted from adipocytes promotes the growth of hepatocellular carcinoma by targeting deubiquitination-related USP7. Oncogene.

[69] Huang G (2020). CircRNA hsa_circRNA_104348 promotes hepatocellular carcinoma progression through modulating miR-187-3p/RTKN2 axis and activating Wnt/β-catenin pathway. Cell Death Dis..

[70] Huang XY (2020). Exosomal circRNA-100338 promotes hepatocellular carcinoma metastasis via enhancing invasiveness and angiogenesis. J. Exp. Clin. Cancer Res..

[71] Su Y (2019). CircRNA Cdr1as functions as a competitive endogenous RNA to promote hepatocellular carcinoma progression. Aging.

[72] Zhang S (2019). CircRNA_0000502 promotes hepatocellular carcinoma metastasis and inhibits apoptosis through targeting microRNA-124. J. BUON.

[73] Zhan W (2020). Circular RNA hsa_circRNA_103809 promoted hepatocellular carcinoma development by regulating miR-377-3p/FGFR1/ERK axis. J. Cell Physiol..

[74] Xu J (2020). CircRNA-SORE mediates sorafenib resistance in hepatocellular carcinoma by stabilizing YBX1. Signal Transduct. Target Ther..

[75] Xu J (2020). N-methyladenosine-modified CircRNA-SORE sustains sorafenib resistance in hepatocellular carcinoma by regulating β-catenin signaling. Mol. Cancer.

[76] Han D (2017). Circular RNA circMTO1 acts as the sponge of microRNA-9 to suppress hepatocellular carcinoma progression. Hepatology.

[77] Wang J, Tan Q, Wang W, Yu J (2020). Mechanism of the regulatory effect of overexpression of circMTO1 on proliferation and apoptosis of hepatoma cells via miR-9-5p/NOX4 axis. Cancer Manag. Res..

[78] Liu Z (2019). CircRNA-5692 inhibits the progression of hepatocellular carcinoma by sponging miR-328-5p to enhance DAB2IP expression. Cell Death Dis..

[79] Fu L (2017). Screening differential circular RNA expression profiles reveals hsa_circ_0004018 is associated with hepatocellular carcinoma. Oncotarget.

[80] Fu L (2017). Hsa_circ_0005986 inhibits carcinogenesis by acting as a miR-129- 5p sponge and is used as a novel biomarker for hepatocellular carcinoma. Oncotarget.

[81] Su Y, Xu C, Liu Y, Hu Y, Wu H (2019). viaCircular RNA hsa_circ_0001649 inhibits hepatocellular carcinoma progression multiple miRNAs sponge. Aging.

[82] Jiang XM (2018). A novel prognostic biomarker for cholangiocarcinoma: circRNA Cdr1as. Eur. Rev. Med. Pharm. Sci..

[83] Li D (2020). Circular RNA CDR1as exerts oncogenic properties partially through regulating miR-641 in cholangiocarcinoma. Mol. Cell Biol..

[84] Xu Y (2019). Elevation of circular RNA circ_0005230 facilitates cell growth and metastasis via sponging miR-1238 and miR-1299 in cholangiocarcinoma. Aging.

[85] Xu Y (2018). Downregulated circular RNA hsa_circ_0001649 regulates proliferation, migration and invasion in cholangiocarcinoma cells. Biochem. Biophys. Res. Commun..

[86] He JH (2018). The CircRNA-ACAP2/Hsa-miR-21-5p/ Tiam1 regulatory feedback circuit affects the proliferation, migration, and invasion of colon cancer SW480 cells. Cell Physiol. Biochem..

[87] Fang G, Ye BL, Hu BR, Ruan XJ, Shi YX (2018). CircRNA_100290 promotes colorectal cancer progression through miR-516b-induced downregulation of FZD4 expression and Wnt/β-catenin signaling. Biochem. Biophys. Res. Commun..

[88] Xie H (2016). Emerging roles of circRNA_001569 targeting miR-145 in the proliferation and invasion of colorectal cancer. Oncotarget.

[89] Yang H (2020). Hypoxia induced exosomal circRNA promotes metastasis of colorectal cancer via targeting GEF-H1/RhoA axis. Theranostics.

[90] Yuan Y, Liu W, Zhang Y, Zhang Y, Sun S (2018). CircRNA circ_0026344 as a prognostic biomarker suppresses colorectal cancer progression via microRNA-21 and microRNA-31. Biochem. Biophys. Res. Commun..

[91] Li XN (2018). RNA sequencing reveals the expression profiles of circRNA and indicates that circDDX17 acts as a tumor suppressor in colorectal cancer. J. Exp. Clin. Cancer Res..

